# Esomeprazole enhances the effect of ionizing radiation to improve tumor control

**DOI:** 10.18632/oncotarget.28008

**Published:** 2021-07-06

**Authors:** Kassidy A. Hebert, Sergio Jaramillo, Wangjie Yu, Min Wang, Ratna Veeramachaneni, Vlad C. Sandulache, Andrew G. Sikora, Mark D. Bonnen, Ananth V. Annapragada, David Corry, Farrah Kheradmand, Raj K. Pandita, Michelle S. Ludwig, Tej K. Pandita, Shixia Huang, Cristian Coarfa, Sandra L. Grimm, Dimuthu Perera, George Miles, Yohannes T. Ghebre

**Affiliations:** ^1^Department of Radiation Oncology, Baylor College of Medicine, Houston, Texas 77030, USA; ^2^Interdepartmental Graduate Program in Translational Biology and Molecular Medicine, Baylor College of Medicine, Houston, Texas 77030, USA; ^3^Department of Otolaryngology – Head and Neck Surgery, Baylor College of Medicine, Houston, Texas 77030, USA; ^4^Department of Head and Neck Surgery, University of Texas MD Anderson Cancer Center, Houston, Texas 77030, USA; ^5^E.B. Singleton Department of Radiology, Texas Children's Hospital and Baylor College of Medicine, Baylor College of Medicine, Houston, Texas 77030, USA; ^6^Department of Obstetrics and Gynecology, Texas Children's Hospital and Baylor College of Medicine, Houston, Texas 77030, USA; ^7^Dan L. Duncan Comprehensive Cancer Center, Baylor College of Medicine, Houston, Texas 77030, USA; ^8^Department of Medicine, Section on Pulmonary and Critical Care Medicine, Center for Translational Research on Inflammatory Diseases, Baylor College of Medicine, Houston, Texas 77030, USA; ^9^Department of Medicine, Section on Pulmonary and Critical Care Medicine, Baylor College of Medicine, Houston, Texas 77030, USA; ^10^Department of Molecular and Cellular Biology, Baylor College of Medicine, Houston, Texas 77030, USA; ^11^Dan L. Duncan Cancer Center, Department of Molecular and Cellular Biology, Baylor College of Medicine, Houston, Texas 77030, USA; ^12^Advanced Technology Cores, Multi-Omics Data Analysis Core, Baylor College of Medicine, Houston, Texas 77030, USA; ^13^Department of Molecular and Human Genetics, Baylor College of Medicine, Houston, Texas 77030, USA

**Keywords:** esomeprazole, proton pump inhibitors, ionizing radiation, radiosensitization, tumor control

## Abstract

The resistance of cancer cells to radiation-based treatment is a major clinical challenge confounding standard of care in cancer. This problem is particularly notable in many solid tumors where cancer cells are only partially responsive to radiation therapy. Combination of radiation with radiosensitizers is able to enhance tumor cell killing. However, currently available radiosensitizers are associated with significant normal tissue toxicity. Accordingly, there is an unmet need to develop safer and more effective radiosensitizers to improve tumor control. Here, we evaluated the radiosensitizing effect of the FDA-approved drug esomeprazole in normal and radioresistant human head and neck squamous cell carcinoma (HNSCC) cells *in vitro*, and in a mouse model of HNSCC. For the *in vitro* studies, we used cancer cell colony formation (clonogenicity) assay to compare cancer cell growth in the absence or presence of esomeprazole. To determine mechanism(s) of action, we assessed cell proliferation and profiled cell cycle regulatory proteins. In addition, we performed reverse phase protein array (RPPA) study to understand the global effect of esomeprazole on over 200 cancer-related proteins. For the *in vivo* study, we engrafted HNSCC in a mouse model and compared tumor growth in animals treated with radiation, esomeprazole, and combination of radiation with esomeprazole. We found that esomeprazole inhibits tumor growth and dose-dependently enhances the cell killing effect of ionizing radiation in wildtype and p53-mutant radioresistant cancer cells. Mechanistic studies demonstrate that esomeprazole arrests cancer cells in the G1 phase of the cell cycle through upregulation of p21 protein and inhibition of cyclin-dependent kinases (Cdks) type 1 (Cdk1) and type 2 (Cdk2). *In vivo* data showed greater tumor control in animals treated with combination of radiation and esomeprazole compared to either treatment alone, and that this was associated with inhibition of cell proliferation *in vivo*. In addition, combination of esomeprazole with radiation significantly impaired repair following radiation-induced DNA damage. Our studies indicate that esomeprazole sensitizes cancer cells to ionizing radiation, and is associated with upregulation of p21 to arrest cells in the G1 phase of the cell cycle. Our findings have significant therapeutic implications for the repurposing of esomeprazole as a radiosensitizer in HNSCC and other solid tumors.

## INTRODUCTION

Radiation therapy is a standard of care approach in the treatment of cancer patients who are medically inoperable or have surgically unresectable tumors. Unfortunately, many solid tumors are only partially responsive to radiation therapy-based interventions. Combination of radiation with radiosensitizers is able to enhance tumor cell killing. However, currently available radiosensitizers, such as cisplatin and other cytotoxic agents, are non-selective and often associated with a plethora of side effects including ototoxicity, infection, hair loss, as well as hematological and cardiovascular complications. Accordingly, there is an opportunity to search and develop safer and effective radiosensitizers. One strategy is to screen among existing drugs including those that are originally approved for non-oncologic indications.

Emerging studies indicate that proton pump inhibitors (PPIs), a class of FDA-approved drug for the treatment of gastroesophageal reflux diseases, have chemosensitizing activity in tumor cells derived from melanoma, colon, breast and ovarian cancers [1–3]. Luciani et al [1], for example, assessed the sensitivity of several treatment-resistant human cancer cell lines upon treatment with the PPIs esomeprazole and omeprazole, and their data shows that pretreatment of the cancer cells with the PPIs resulted in order of magnitude reduction in the half maximal inhibitory concentration (IC_50_) values for the chemotherapeutic agents cisplatin, vinblastine and 5-fluorouracil compared to no PPI control. Additionally, their *in vivo* study demonstrated that pretreatment of engrafted tumors with PPIs increased sensitivity of the tumor cells to cisplatin resulting in significant reduction in tumor weight. Similarly, several other studies in mice, cats and dogs have demonstrated significant improvements in the sensitivity of tumor cells derived from kidney cancer, gastric cancer, esophageal cancer, adenocarcinoma, osteosarcoma and lymphomas to several anticancer drugs upon pretreatment with PPIs [4–10]. In addition, studies in companion animals with spontaneously occurring tumors have shown increased tumor response upon combination of the PPI lansoprazole with metronomic chemotherapy [11]. Some of the proposed mechanisms for the chemosensitizing effect of PPIs include the effect of the drug on cancer cell invasion, migration and adhesion; buffering the acidic tumor microenvironment; as well as increased chemotherapeutic drug uptake by the tumor cells [2, 12, 13].

In line with the increased chemosensitizing effect of PPIs in solid tumor-derived cancer cells in preclinical models, clinical studies also reported that PPIs are associated with beneficial outcomes in cancer patients including these with refractory disease [14–16]. In addition, high doses of PPIs have been safely administered to cancer patients to achieve plasma drug concentrations of about 100 μM [17]. Collectively, the wide margin of safety of PPIs in cancer patients, and their chemosensitizing effect in preclinical models, as well as in humans provoked us to address the question of whether they can be combined with radiation to enhance antitumor effect. Accordingly, we carried out molecular, cell biological and *in vivo* experiments at clinically achievable drug concentrations to evaluate the effect of a prototype PPI, esomeprazole, on the proliferation, cell cycle, colony formation and DNA damage response in two human HNSCC cell lines (HN30 and HN31). The HN31 cell line carries a disruptive mutation (C176F) in the *TP53* gene and is relatively radioresistant compared to the isogenic wildtype *TP53* expressing HN30 cell lines [18]. *In vivo*, we evaluated the efficacy of esomeprazole, alone or in combination with radiation, in controlling the growth of cancer cells in a syngeneic mouse model of HNSCC.

## RESULTS

### Esomeprazole possesses anticancer activity

**Figure 1:**
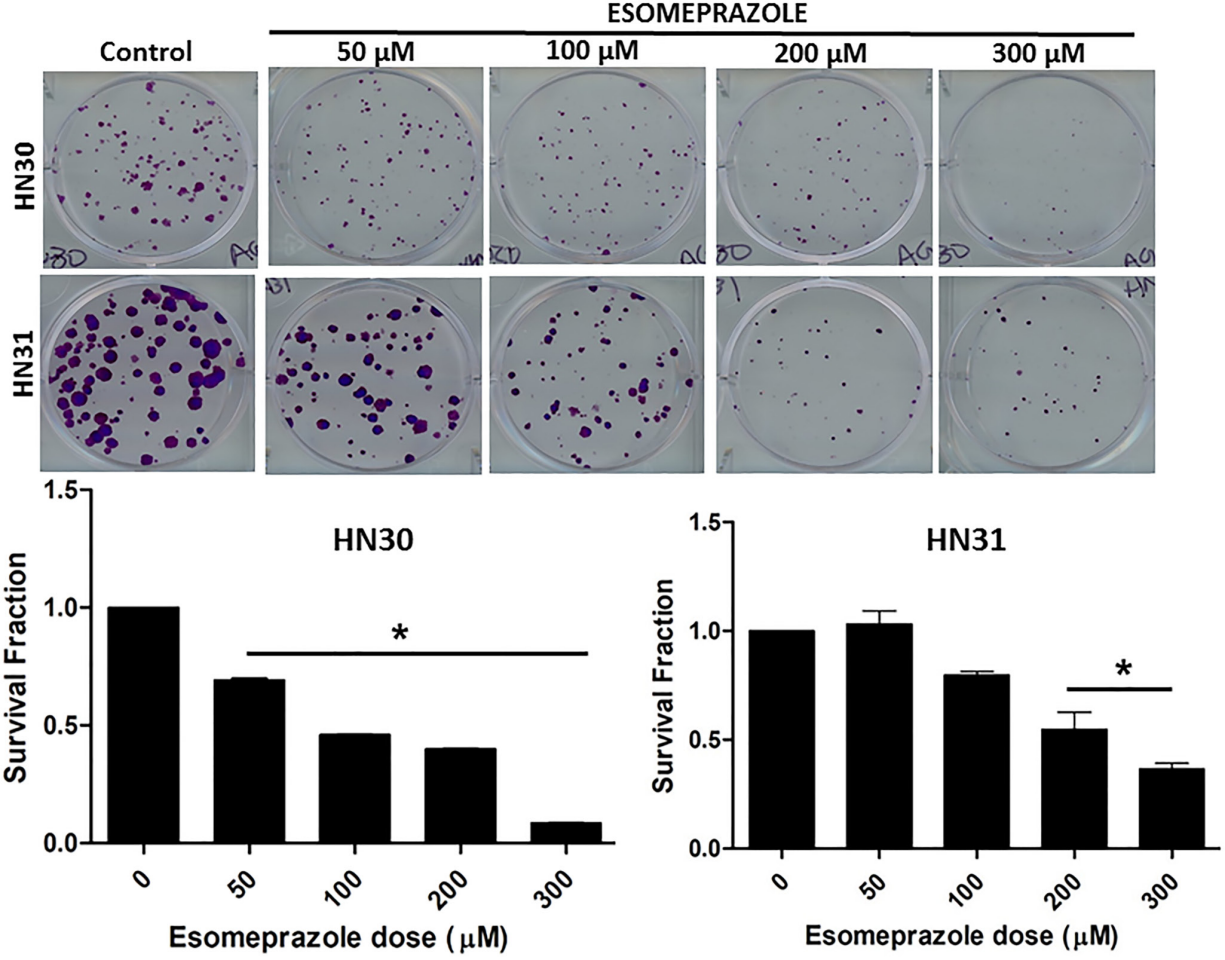
Esomeprazole inhibits the growth of head and neck cancer cells. Wild type (HN30) and p53-mutant (HN31) head and neck squamous cancer cells were cultured in six-well plates and treated with vehicle (control) or various concentrations of esomeprazole (50–300 μM) for 24 hours. Cells were allowed to form colonies for 2 weeks prior to staining with crystal violet (0.05%). The bar graphs show quantification of the data. Data is representative of eight replicate experiments (^*^ *p* < 0.05 vs control).

Our colony formation assay demonstrated that esomeprazole possesses significant anticancer activity. Brief (24 hours) incubation of the head and neck cancer cells HN30 and HN31 at the beginning of the assay showed durable inhibition of growth for at least 2 weeks (Figure 1). The data also shows that despite the differences in the functional status of p53, and the differential sensitivity of the cells to anticancer drugs [19], HN30 and HN31 cells were equally sensitive to inhibition by esomeprazole. Notably, the anticancer effect of esomeprazole in the head and neck cancer cells was reproduced in breast (MCF-7) and lung (NCI-H460) cancer cells (Supplementary Figure 1). Intriguingly, the effect of esomeprazole on the growth of the head and neck, breast and lung cancer cells was significantly enhanced by extending the incubation time beyond 24 hours (data for HN30 is shown in Supplementary Figure 2). As expected, the anticancer effect of esomeprazole was reproduced with another PPI, lansoprazole, that shares common chemical structure (Supplementary Figure 3). However, this effect could not be reproduced with alternative antacids, the H2RAs, ranitidine or famotidine (Supplementary Figure 4), that have distinct chemical structure than esomeprazole.

### Esomeprazole enhances the effect of radiation to improve tumor control: *in vitro* and *in vivo* evidence

**Figure 2:**
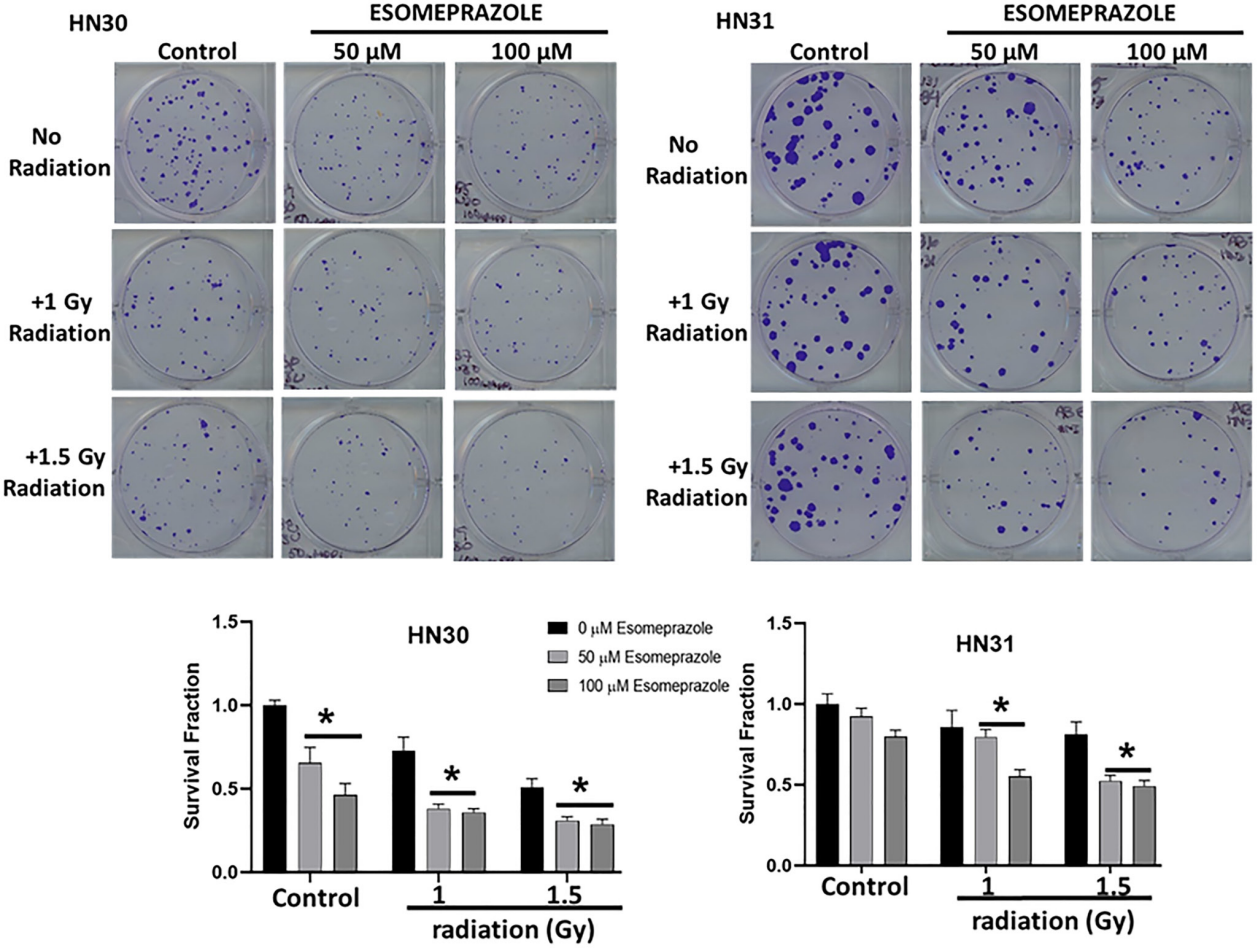
Esomeprazole enhances the effect of radiation to improve tumor control *in vitro*. HN30 and HN31 cells were cultured in six-well plates in replicates. Some of the wells were subjected to ionizing radiation (X-rays; 1–1.5 Gy) in the absence or presence of esomeprazpole (50–100 μM for 24 hours). Cancer cell colonies were stained at 2 weeks for comparison. The bar graphs show decreased survival fraction of HN30 and HN31 cells following radiation and/or esomeprazole treatment in comparison to controls. Data is representative of six replicate experiments (^*^ *p* < 0.05 vs control).

**Figure 3:**
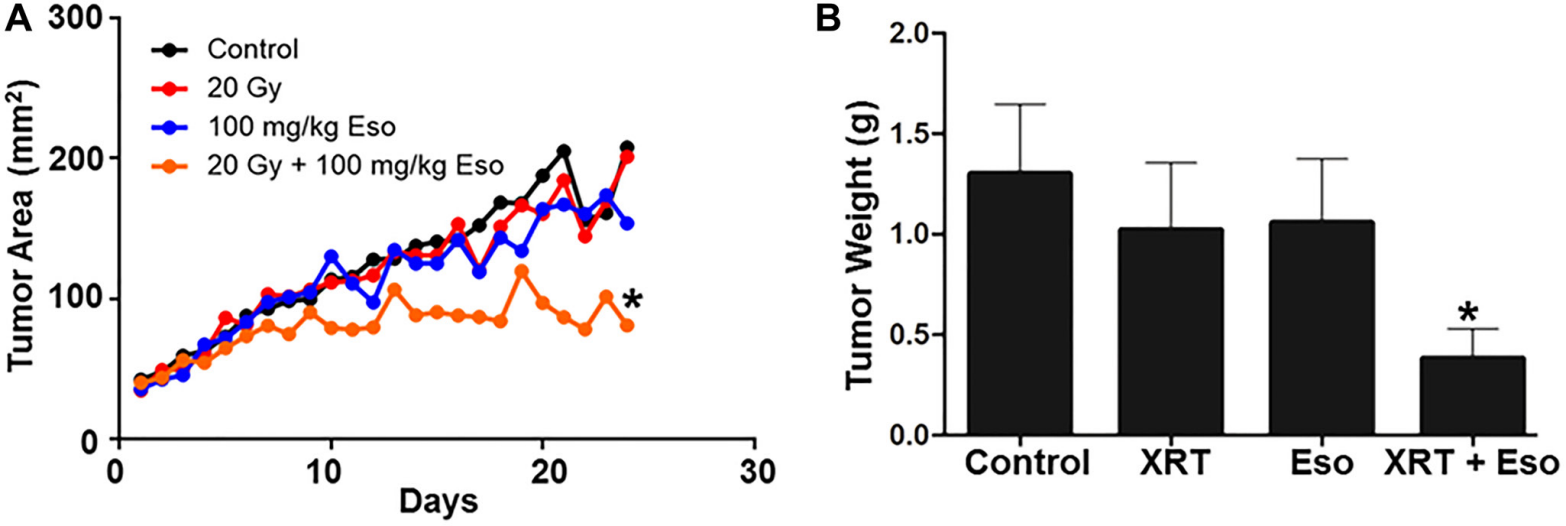
Esomeprazole enhances the effect of radiation to improve tumor control *in vivo*. C57BL/6J mice were subcutaneously injected with mouse oropharyngeal epithelial cells (MEER; 8 × 10^5^) transformed with oncogenes. The tumor was allowed to reach 40 mm^2^ before administering vehicle (water), esomeprazole (100 mg/kg), radiation (20 Gy), or combination of radiation and esomeprazole. In (**A**) growth kinetics of the tumor was measured every day using caliper. In (**B**) the tumor weight is shown for the control (1.302 ± 0.35), esomeprazole (1.057 ± 0.32), radiation (1.023 ± 0.33), and combination of radiation and esomeprazole (0.3838 ± 0.15). Average data is shown from 6–8 animals/group (^*^ *p* < 0.05 vs control or monotherapy).

**Figure 4:**
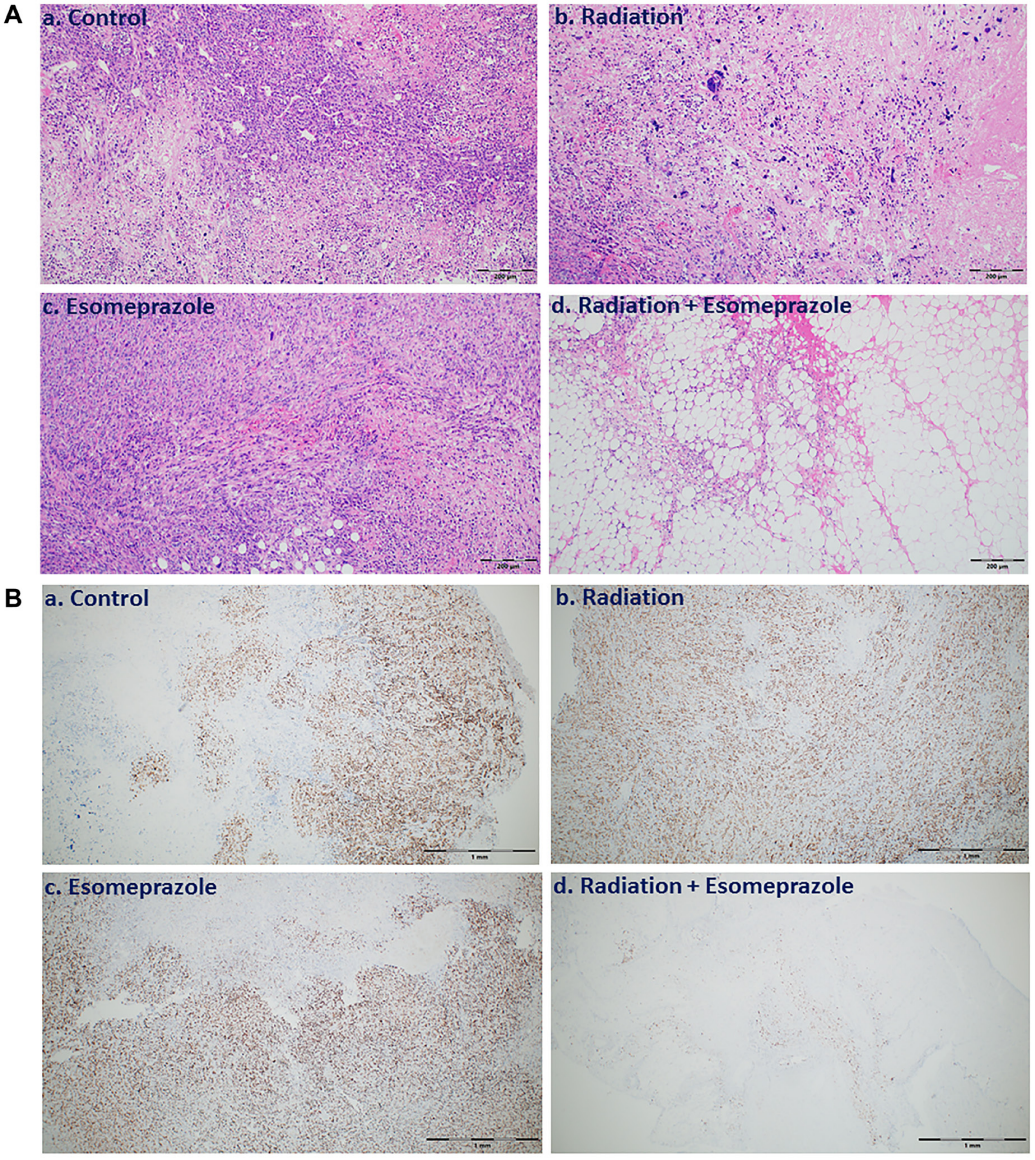
Combination of esomeprazole and radiation reduces cancer cell growth and proliferation. Tumor tissue explanted from vehicle, esomeprazole, radiation, or combination of radiation and esomeprazole were stained for Hematoxylin and Eosin (H&E) for overall tissue architecture, and immunohistochemistry for the cell proliferation marker Ki67. (**A**) shows reduction in viable tumor in the explanted tissues from combination of radiation and esomeprazole treated animals (d) compared to the poorly differentiated tumor seen in the control (a), and monotherapy treated animals (b and c). (**B**) Ki67 immunostaining shows inhibition of cell proliferation by radiation and esomeprazole combination compared to all other groups. At least 6 non-overlapping fields per sample were assessed. Scale bar shown is 200 μm for the H&E images, and 1 mm for the Ki67 images.

Encouraged by the anticancer effect of esomeprazole, we sought to evaluate whether esomeprazole can be combined with ionizing radiation to sensitize cancer cells. Our *in vitro* colony formation assay demonstrated that esomeprazole dose-dependently enhanced the killing effect of radiation in both HN30 and HN31 cells (Figure 2). The radiosensitizing effect of esomeprazole in the head and neck cancer cells was reproduced in breast and lung cancer cells (Supplementary Figure 5). Our *in vivo* study in a mouse model of HNSCC demonstrated that the effect of radiation on tumor control can be significantly enhanced with esomeprazole as shown by reduction in the tumor area of combined radiation and esomeprazole treated animals compared to treatment with radiation alone (Figure 3A). The post-necropsy review showed that the animals treated with the combination of esomeprazole and radiation had smaller composite tumor mass compared to radiation- or esomeprazole- alone groups (Figure 3B). Histopathological analysis of the explanted tumor tissue demonstrate that the combination treated group had little presence of tumor compared to the poorly differentiated tumor observed in the control or monotherapy treated animals (Figure 4A). Immunohistochemical staining for the proliferation marker Ki67 qualitativly confirmed the reduction in the number of proliferating tumor cells between the combination of esomeprazole and radiation treated group and all other groups (Figure 4B).

### Esomeprazole inhibits cancer cell proliferation

**Figure 5:**
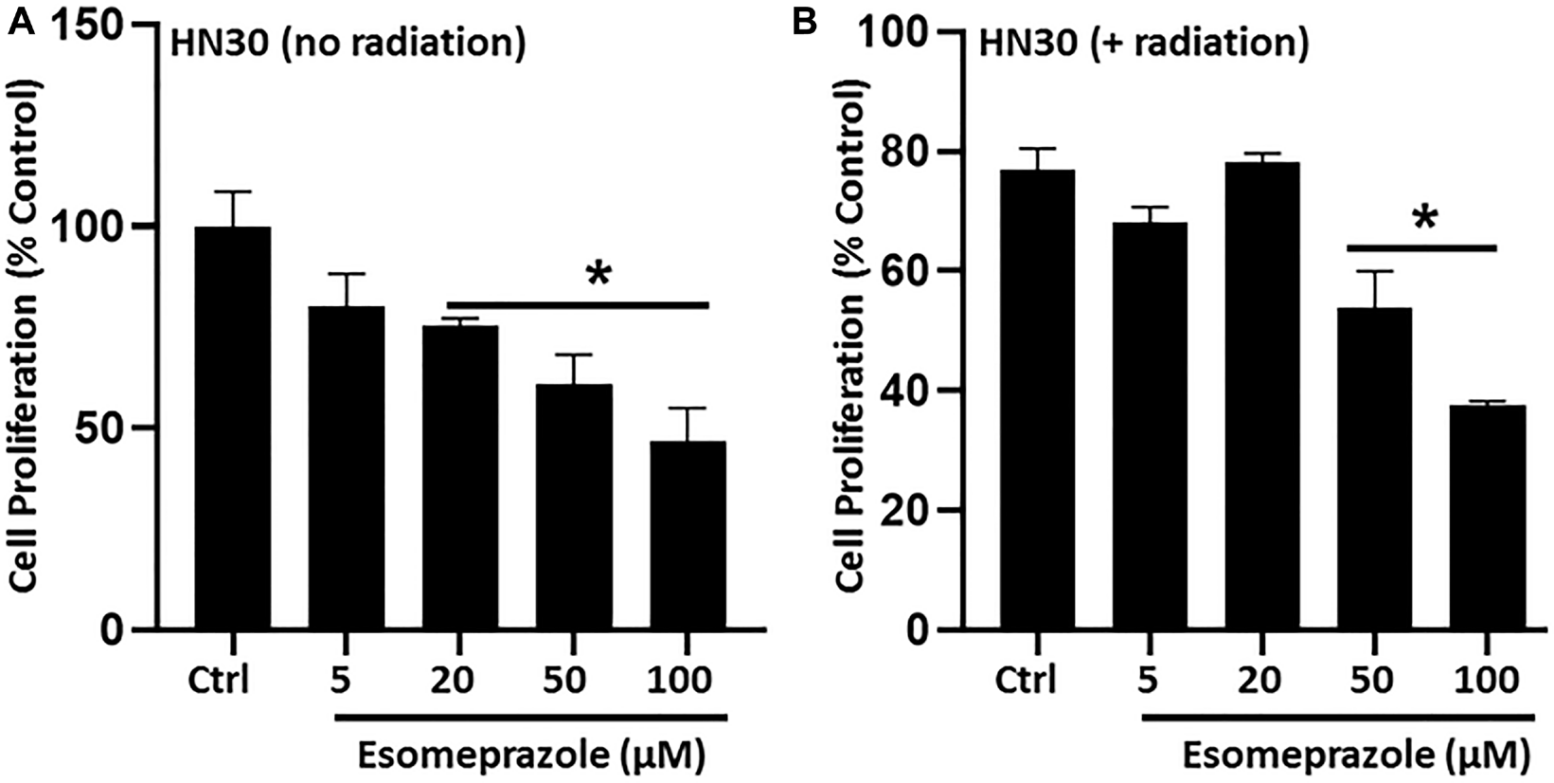
Esomeprazole inhibits cancer cell proliferation. HN30 cells (3 × 10^3^ cells) were seeded in 96-well plates and synchronized by serum starvation prior to stimulation to induce proliferation in the presence of vehicle (water) or esomeprazole for 24 hours without (**A**) or with (**B**) radiation (X-rays; 1 Gy). The incorporation of 5-bromo-2-deoxyuridine (BrdU) into the DNA of proliferating cells was determined by absorbance (OD 450 nm) using a spectrophotometer. The readouts were compared among the groups to assess the differential effect of esomeprazole on cell proliferation. Data is Mean ± SEM from triplicate experiments. ^*^ *p* < 0.05 compared to vehicle control (Ctrl).

Abnormal proliferation of cancer cells is a major contributor to carcinogenesis. In this study, we found that esomeprazole inhibited the proliferation of cancer cells in the absence or presence of radiation as shown by decreased incorporation of BrdU into the DNA of proliferating cells (Figure 5). In the absence of radiation, esomeprazole inhibited the proliferation of HN30 cells by about 50% at 100 μM (Figure 5A). Similarly, cell proliferation was modestly inhibited by radiation alone, and combination with esomeprazole enhanced control of the proliferating cells. As shown in Figure 5B, co-treatment of HN30 cells with esomeprazole and radiation reduced the percentage of proliferating cells by more than 30% compared to radiation only treated cells. Notably, inhibition of cancer cell proliferation by esomeprazole is not due to non-specific cytotoxicity since treatment of cells with esomeprazole at or above concentrations used in the proliferation assay neither affected cell viability nor compromised the integrity of cell membrane as confirmed by lack of change in the release of lactate dehydrogenase (LDH) from intracellular compartments into the conditioned media following esomeprazole treatment [20].

### Esomeprazole arrests cancer cells at G0/G1 phase to block proliferation

**Figure 6:**
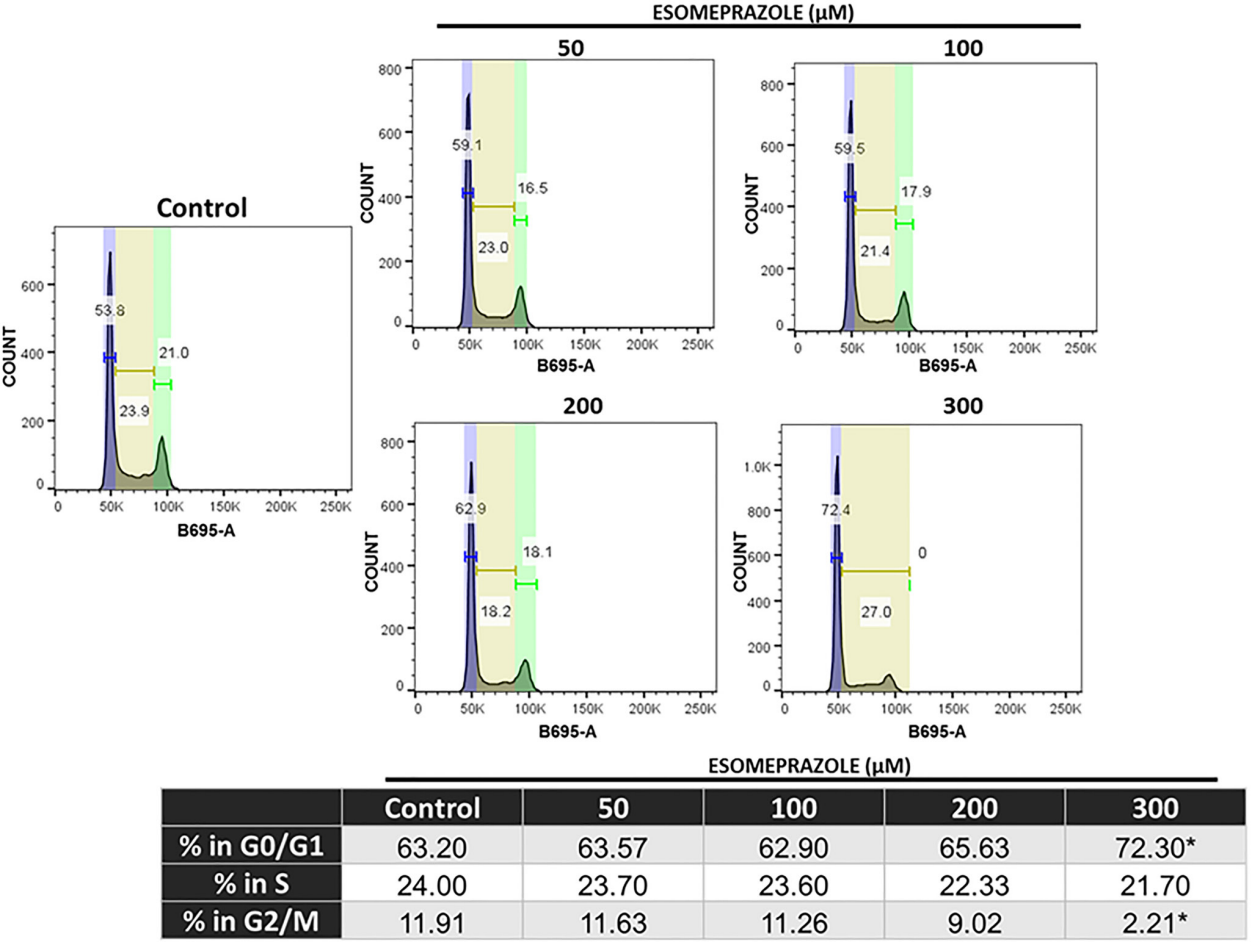
Esomeprazole arrests cells in the G1/G0 phase of the cell cycle. HN30 cells were treated with vehicle (water) or esomeprazole for 24 hours. Cells were harvested and stained with propidium iodide (B695-A) and ran through a flow cytometer. Data was analyzed using FlowJo, and the averaged cell counts in each group are shown. ^*^ *p* < 0.05 compared to vehicle control.

Fluorescence-activated cell sorting (FACS) data show that treatment of cancer cells with esomeprazole significantly arrests growth in the early phases (G0/G1) of the cell cycle at 300 μM (Figure 6). The study also shows that the proportion of cells in the G0/G1 phase at the highest tested concentration of esomeprazole was equivalent to the combined number of cells in the G0/G1 and G2/M phases of the cell cycle in the vehicle-treated group (Table in Figure 6).

### Esomeprazole augments radiation-induced double-strand DNA breaks to enhance antitumor activity

Given that generation of DNA strand breaks is one of the principal mechanisms by which ionizing radiation kills cancer cells, we studied whether the radiosensitizing effect of esomeprazole is, at least in part, due to sustaining the DNA damage induced by radiation. Notably, immunofluorescence staining of the histone protein and key molecular marker of DNA damage, γ-H2AX, showed that co-treatment of irradiated cancer cells with esomeprazole significantly enhanced accumulation of phosphorylated H2AX foci in the nuclei (Supplementary Figure 6).

### Combination of esomeprazole with radiation enhances residual chromosome aberrations

To determine whether increased level of γ-H2AX in cells treated with esomeprazole is due to the presence of residual DNA damage after exposure to ionizing radiation, we analyzed whether esomeprazole impacts chromosomal damage repair after irradiation. To analyze G1-type aberrations, exponentially-growing cells were treated with esomeprazole in the absence or presence of radiation, and metaphase cells were collected as described [21]. G1-type aberrations including dicentrics, fragements, breaks and gaps were measured and cells treated with esomeprazole were found to have significantly higher chromosomal aberrations compared to control cells (Supplementary Figure 7). Similarly, S-phase and G2-type chromosomal aberrations were analyzed and showed no differences in S-phase or G2-specific chromosome and chromatid aberrations between esomeprazole treated and untreated cells (data not shown). These results suggest that higher frequency of γ-H2AX is likely due to unrepaired DNA damage during G1-phase of the cell cycle.

### Esomeprazole differentially regulates a number of cancer-related proteins

The RPPA study revealed that esomeprazole differentially regulates several cell cycle-related proteins including p21, p300, ULK1, and cyclin C. In addition, treatment with esomeprazole phosphorylated important signaling proteins including RAC-alpha serine/threonine protein kinase (AKT1), AKT1 substrate 1 (AKT1S1, PRAS40), and extracellular signal-regulated protein kinase (ERK1/2) (Supplementary Table 1 and Supplementary Figure 8). Complete profile of the esomeprazole regulated proteins is shown in Supplementary Table 1. Our validation study by Western blot shows that p21 and ULK1 are dose-dependently upregulated by esomeprazole in the absence or presence of radiation (Supplementary Figure 9A). As expected, the p21 target kinases Cdk1 and Cdk2 were downregulated by esomeprazole (Supplementary Figure 9B).

## DISCUSSION

### Esomeprazole possesses anticancer activity

Emerging studies indicate that proton pump inhibitors (PPIs) such as esomeprazole favorably regulate cancer cell growth, metastasis, autophagy and sensitization to common chemotherapeutic drugs [1, 12, 22–25]. For example, treatment of breast cancer cells that lack expression for the receptors of estrogen, progesterone and human epidermal growth factor receptor 2 (HER2), commonly known as “triple-negative”, with esomeprazole significantly and dose-dependently inhibited their growth [10]. The authors attributed the antitumor effect of esomeprazole to increased intracellular acidification. Similarly, several studies have reported anticancer activity of PPIs in cancers of the digestive system [26–28], melanoma [29, 30], lymphoma [31, 32], leukemia [31, 33], myeloma [34] and osteosarcoma [8].

Notably, many of these studies have identified PPI targets that are critical for the growth, proliferation and metastasis of cancer cells. For example, in gastric cancer cells, PPIs have been shown to inhibit the expression and nuclear translocation of hypoxia-inducible factor 1 α (HIF-1α), Wnt/β-catenin signaling, and the IL-6/STAT3 signaling pathway [7, 35, 36]. In addition, several studies have attributed the anticancer effect of PPIs to regulation of intra-tumoral pH secondary to inhibition of proton pumps including the H^+^/K^+^-ATPase [2].

Our biological studies in primary human cancer cells derived from the pharynx (HN30), lymph-node (HN31), breast (MCF-7) and lungs (NCI-H460) of cancer patients demonstrate that the PPI esomeprazole inhibits the growth of these cancer cells in a dose and time dependent manner (Figures 1–3 and Supplementary Figures 1, 2 and 5). This effect was reproduced by another PPI, lansoprazole, that shares common benzimidazole core structure (Supplementary Figure 3). However, neither members of the H2RAs, ranitidine or famotidine, could reproduce the effect of esomeprazole on tumor growth (Supplementary Figure 4) suggesting that the anticancer effect of PPIs *in vitro* is unlikely due to non-specific ‘buffering’ effect of antacids. In addition, our clonogenic assay studies have been conducted under physiological pH (pH = 7.6) indicating that low pH-based activation of PPIs is not a requirement for their anticancer activity. Our mechanistic studies revealed that esomeprazole upregulates the expression of p21 (Supplementary Figure 9A); a Cdk inhibitor that targets the activity of several Cdks including Cdk1, Cdk2 and Cdk4/6 to arrest cells in the G1 phase of the cell cycle [37]. Accordingly, we found that the expression of both Cdk1 and Cdk2 was inhibited by esomeprazole in HN30 head and neck cancer cells (Supplementary Figure 9B). The two kinases are important for cell cycle progression including spindle assembly, sister chromatid alignment, chromatid segregation and entry into the mitosis (M) and growth (G1) phases of the cell cycle. In line with this, our FACS study demonstrated that esomeprazole arrested HN30 cells in the G1 phase and inhibited entry into the S phase where DNA replication takes place. As a result, the number of cells entering the G2 phase is severely impaired by esomeprazole (Figure 6). Although this effect was only observed at the 300 μM drug concentration, it is consistent with the antiproliferative effect of the drug (Figure 5). Nonetheless, the cell cycle data is indicative of additional mechanisms of action that are potentially involved at high drug concentrations.

### Esomeprazole enhances the effect of radiation to control tumor growth *in vivo*

Encouraged by the *in vitro* findings that show anticancer effect of esomeprazole, we evaluated the efficacy of the drug in a mouse model *in vivo*. Our data shows that esomeprazole alone had some effect on tumor growth and combination with radiation had significant effect in reducing tumor growth compared to radiation alone (Figure 3). Histopathological studies corroborated the macroscopic observations in that there was little tumor and significantly fewer cells expressing Ki67; a protein associated with tumor cell proliferation and growth (Figure 4).

### Esomeprazole radiosensitizes cancer cells *in vitro*

Our microscopy study shows that esomeprazole alone increases the expression of γ-H2AX foci, and this effect is enhanced in the presence of ionizing radiation (Supplementary Figure 6). The effect of esomeprazole on DNA damage is in line with an earlier study that reported induction of γ-H2AX by another PPI, pantoprazole, in prostate cancer cells co-treated with docetaxel [25]. However, this is the first study, to our knowledge, to demonstrate increased DNA damage by esomeprazole in the context of radiation. Although the exact mechanism of action for the effect on DNA damage remains unclear, our chromosomal aberrations study demonstrates that esomeprazole impairs DNA damage repair (Supplementary Figure 7). Therefore, it is plausible that PPI-induced electrophilic stress [38] may impair DNA damage repair following exposure to ionizing radiation. Intriguingly, the radiosensitizing effect of esomeprazole was achieved under physiological pH, without the need to be exposed to acidic conditions.

### Esomeprazole regulates several cancer-related proteins

Consistent with our cell biological study that shows favorable effect of esomeprazole in controlling cancer cell growth, the RPPA data revealed that esomeprazole regulates critical cell cycle proteins such as p21, p300, cyclin C, and ULK1 (Supplementary Figures 8, 9, and Table 1). For example, upregulation of p21 is expected to arrest cells in the G0/G1 phase of the cell cycle to prevent entry into the S-phase for DNA replication [39].

In conclusion, esomeprazole, a common FDA-approved drug for the treatment of acid reflux, has anticancer activity that dose-dependently limits the growth of primary human cancer cells derived from various tissue sources. Intriguingly, combination of esomeprazole with ionizing radiation significantly enhances tumor control *in vitro* and *in vivo*. Mechanistically, the anticancer effect of esomeprazole appears to in part be due to its antiproliferative activity that involves induction of p21; a key cell cycle protein and a potent cyclin-dependent kinase inhibitor (CKI). Induction of p21 with esomeprazole resulted in the inhibition of cyclin-dependent kinases (Cdks) to arrest proliferating cancer cells from proceeding with critical cell cycle events including DNA replication and mitosis (Supplementary Figures 9 and 10). Consequently, the ability of the cancer cells to clonally expand *in vitro* and *in vivo* is significantly inhibited (Figures 1–3). These targeted molecular activities and the buffering effect on tumor microenvironment [40, 41] may enable PPIs to exert pleiotropic anticancer activity. Paradoxically, PPIs are selective in sensitizing cancer cells to chemoradiation therapy (e.g. Figures 2–3 and [20]) but are protective of normal tissue [42]. This unique property should be leveraged for the evaluation and rapid development of esomeprazole and its analogs in clinical studies. However, the potency of esomeprazole analogs or other PPIs should be empirically tested *in vitro,* and the most promising candidates should be evaluated in animal models and early phase clinical trials. In this regard, previous studies have documented differences in the anticancer activity of PPIs [43]. Emerging studies indicate that PPIs including esomeprazole are associated with favorable outcomes in the prevention of cancer or treatment of cancer patients [14, 44–46]. Papagerakis et al [14] studied a cohort of 596 patients with head and neck squamous cell carcinomas (HNSCC) and reported that patients administered with PPIs in addition to conventional care had significantly longer survival compared to patients on conventional treatment of surgery with or without chemoradiotherapy. In addition, Wang et al [44] reported increased progression-free survival and overall survival in colorectal cancer patients who received standard of care supplemented with PPIs compared to standard of care alone. Currently, there are completed or ongoing clinical trials evaluating PPIs as adjuvants in cancer patients (e.g. NCT01069081). For example, a recent study presented at the 2020 American Society of Clinical Oncology (ASCO) reported that administration of PPIs with chemotherapy to triple-negative breast cancer (TNBC) patients was associated with significant pathologic complete response (pCR) rate without adding toxicity (clinical trial # NCT02595372) [47]. Although the doses of PPI required to achieve plasma concentration of about 100 μM for radiosensitizing activity is higher than the standard antacid dose, it is safely achievable in cancer patients. In this regard, a recent phase I study in patients with advanced solid tumors demonstrated that up to 360 mg of PPI can be safely administered to ahieve plasma concentration of about 100 μM [17]. Therefore, PPIs have tremendous potential to be repurposed as anticancer agents, chemosensitizers, and/or radiosensitizers to improve tumor control. The anticancer and chemosensitizing activity of PPIs and their potential to be repurposed for clinical use has recently been discussed [2, 3, 48]. Future studies are expected to similarly test and develop the radiosensitizing effect of PPIs.

## MATERIALS AND METHODS

### HNSCC colony formation assay

Wildtype (HN30) and p53-mutant (HN31) primary human head and neck squamous carcinoma cells, as well as mouse oropharyngeal epithelial cells (MEER) transformed with oncogenes [49] were used to test the anticancer/radiosensitizing effect of esomeprazole. The cells were cultured under standard cell culture conditions including 37^°^C/5%CO_2_ in Dulbecco’s Modified Eagle’s Medium (DMEM; Sigma; cat # 51435C) supplemented with 10% fetal bovine serum (FBS; Sigma; cat # 12103C), 1% penicillin-streptomycin (Sigma; cat # P4333), 1mM sodium pyruvate (Millipore; cat # TMS-005-C), non-essential amino acids (1X; Sigma; cat # M7145) and vitamins (1X; ThermoFisher Scientific; cat # 11120052). For the MEER cells, the DMEM was supplemented with Ham’s F-12 nutrient mix (ThermoFisher Scientific; cat # SH30026.01) fortified with growth factors. The cell number was counted to seed 400 cells/well of HN30, and 200 cells/well of HN31 in 6-well plates. The cells were incubated overnight prior to treatment with vehicle (water) or various concentrations of esomeprazole (1–300 μM) for 24–96 hours. In some plates, the cells were treated with 1–2 Gray of radiation to study the efficacy of combining esomeprazole with radiation in controlling cancer cell growth. The cells were allowed to form colonies for two weeks, and the number of colonies among the various groups were compared after staining the plates with 0.05% crystal violet in 4% formaldehyde for 60 minutes. Finally, the number of cancer cell colonies in each well were counted and the survival fraction was calculated by dividing the number of colonies in treated wells by the number of colonies in respective control wells. As a pH control and to address whether the effect of esomeprazole on cancer cells is due to ‘buffering’ effect of the acidic tumor microenvironment, we used other non-PPI antacids such as the histamine H2-receptor antagonists (H2RAs) ranitidine and famotidine. To evaluate whether the anticancer/radiosensitizing effect of esomeprazole extends to cancer cells derived from sites other than the head & neck area, we used the MCF-7 breast cancer cells (ATCC; cat # HTB-22) and the NCI-H460 lung cancer cells (ATCC; cat # HTB-177).

### Proliferation of HNSCC

To evaluate whether esomeprazole controls cancer cell growth by inhibiting proliferation, HN30 and HN31 cells were cultured under standard cell culture conditions as described above. The cells were grown to about 80% confluency in DMEM and subsequently seeded in 96-well plates (3 × 10^3^ cells/well) for BrdU cell proliferation assay (Millipore; cat # 2750). The next day, the cells were synchronized by culturing in serum-free media for 2 hours and in low serum (0.1%) media for another 22 hours. On day 3, the cells were incubated in fully-supplemented DMEM in the presence or absence of esomeprazole (1 – 300 μM) for 24 hours. Finally, the cells were incubated with 20 μL of 5-bromo-2-deoxyuridine (BrdU; 1:500) for 24 hours, and the incorporation of BrdU into the proliferating cells’ DNA was measured spectrophotometrically, as per the protocol provided in the kit. Normalized absorbance readouts were compared among the groups to assess the differential effect of esomeprazole on cancer cell proliferation.

### Cell cycle analysis

To determine whether esomeprazole inhibits cancer cell proliferation through regulation of cell cycle, we used flow cytometry to profile HN30 cells across the different stages of the cell cycle. For this, we seeded 90 × 10^3^ cells in 25 mm^2^ flasks in DMEM. The cells were allowed to grow for 3 days at 37^°^C/5%CO_2_ cell culture incubator. On day 4, the cells were synchronized as described above. Next, the cells were incubated in fully-supplemented media in the presence or absence of esomeprazole (1–300 μM) for 24 hours. Subsequently, the cells were harvested and counted. One million cells were washed in 5 mL PBS in centrifuge tubes and thoroughly suspended in 0.5 mL PBS to minimize cell aggregates. The cells were fixed in 70% ethanol for 2 hours and then washed in 5 mL PBS for 1 minute. Finally, the PBS was removed by centrifugation at 200 × g, and the cells were resuspended in 1 mL propidium iodide (Sigma; cat # P4864)/Triton X-100 staining solution containing RNase A in polypropylene tubes. Finally, the cells were analyzed using BD FACSCanto II, and FlowJo software (BD Life Sciences; Franklin Lakes, NJ) was used to profile cells into G0/G1, S and G2/M phases of the cell cycle.

### Assessment of DNA strand breaks

In order to examine whether esomeprazole radiosensitizes cancer cells through potentiation of radiation-induced DNA strand breaks, we performed high throughput immunofluorescence staining to assess the phosphorylation status of the known double-strand DNA break marker H2AX [50]. For this, we seeded HN30 and HN31 cells in 384-well plates at 800 cells/well and 600 cells/well respectively. The next day, the cells were treated with radiation alone (1 Gray); esomeprazole alone (1–300 μM); or combination of esomeprazole with radiation. Subsequently, the cells were fixed with paraformaldehyde (4%), washed with PBS. The samples were then washed with 0.1% TBST (Tris-buffered saline, 0.1% Tween 20) prior to blocking non-specificity with 5% non-fat milk and 0.02% sodium azide for 1 hour at room temperature. Next, the samples were stained for phosphorylated H2AX (γ-H2AX) by incubating with phospho-histone H2AX (Ser139) rabbit monoclonal antibody (Cell Signaling; cat # 9718; 1:500) overnight at 4^°^C. Subsequently, the primary antibody was removed, the samples were washed with blocking buffer and incubated with Alexa Fluor 488 conjugated goat anti-rabbit secondary antibody (ThermoFisher; cat # A-11008) for 30 minutes at room temperature. Next, the secondary antibody was removed, the samples were washed with 0.1% TBST prior to counterstaining the nuclei with DAPI for 1 minute at room temperature. Finally, the DAPI was removed and the samples were incubated in 0.1% TBST for imaging. Multiple fields per sample were scanned for γ-H2AX staining using Optical Biosystems StellarVision (SV20) microscopy. The total number of γ-H2AX foci were counted in all the wells and the replicates were averaged for comparison.

### Chromosomal aberrations studies

To evaluate whether esomeprazole affects chromosomal integrity of cancer cells, we checked for frequencies of chromosomal breaks during mitosis. HN30 cells were prepared using a standard assay [21], and treated with esomperazole or combination of esomeprazole and radiation. Metaphase cells were harvested at 1.5, 5 and 12 hours following esomeprazole/radiation treatment and chromosome aberrations including dicentrics, centric rings, interstitial deletions/acentric and radials were scored as described previously [51, 52].

### Reverse phase protein array (RPPA)

To understand the molecular basis by which esomeprazole controls cancer cell growth, we utilized high throughput RPPA technology [53, 54] to screen over 200 cancer-related proteins that were inventoried by our Antibody-Based Proteomics Core Facility. In this experiment, we treated HN30 cells with concentrations of esomeprazole that showed anticancer activity in our colony formation and cell proliferation assays (i.e., 50–100 μM). The cells were treated with vehicle or esomeprazole for 24 hours and total protein was isolated for analysis. Briefly, equal amount of protein was spotted in triplicates onto nitrocellulose-coated glass slides and a panel of pre-validated primary antibody targeted at various cellular processes including cell cycle and DNA strand break repair were hybridized with the cellular proteins. Each slide was incubated with a specific primary antibody in the presence of no antibody control. Antibody binding was detected using a biotinylated secondary antibody followed by streptavidin-conjugated fluorophore (IRDye680; LI-COR Biosciences, Lincoln, NE). Next, total protein content of each spotted lysate was fluorescently determined by staining with Sypro Ruby Protein Blot Stain following the purveyor’s protocol (Molecular Probes, Eugene, OR). Subsequently, the fluorescently-labeled slides, along with corresponding negative controls, were scanned on a GenePix 4400 AL scanner at an appropriate PMT to obtain optimal signal. At this point, the images were analyzed with GenePix Pro 7.0 (Molecular Devices, San Jose, CA) and total fluorescence signal intensities of each spot were obtained after subtraction of background signal for each slide, and were normalized for variation in total protein, background, and non-specific labeling as described [55]. Finally, the-background-subtracted signal intensity was subtracted by the corresponding signal intensity of the negative control slide for each arrayed spot and normalized to the corresponding signal intensity of total protein for that spot. The quality of data was assessed, and median value of the triplicate experiments (i.e., normalized signal intensity) was used in each of the samples for statistical analysis. Antibodies with median value greater than 100 in at least one experimental group were selected for further analysis. Significantly changed proteins among the groups were determined using Student’s *t*-test (significant for *p* < 0.05) and fold-change of at least 1.5. For validation of hits, the expression of p21 and Unc-51 like autophagy activating kinase (ULK1) was verified by Western blot using rabbit anti-p21 and anti-ULK1 monoclonal antibody (Cell Signaling; cat # 2947; and cat # 14202 respectively). In addition, the p21 target kinases, cyclin-dependent kinase 1 (Cdk1) and cyclin-dependent kinase 2 (Cdk2), were probed using rabbit- anti-Cdk1 (Abcam; cat # ab131450) and anti-Cdk2 (Cell Signaling; cat # 2546) antibody respectively.

### *In vivo* study: mouse model of HNSCC

To validate the radiosensitizing effect of esomeprazole observed in our *in vitro* assays, we performed *in vivo* efficacy study in a mouse model of HNSCC. For this, we used immunocompetent mouse model where C57BL/6J mice were subcutaneously implanted with syngeneic mEER cells established to model HNSCC [49]. The animals were grouped into control (no radiation; no esomeprazole; *n* = 6), radiation (*n* = 6), esomeprazole (*n* = 8), or radiation plus esomeprazole (*n* = 8) groups. All the animals were subcutaneously injected with 1 × 10^6^ mEER cells into the left flank. Once the engrafted tumor reached 65 mm^2^, 10 Gray of radiation was delivered to the tumor in the radiation alone, and radiation plus esomeprazole groups at a dosimetric rate of 1.5 Gray per minute. At this point, daily schedule of intratumoral water (control) or esomeprazole treatment (100 mg/kg) was started in the control, esomeprazole alone, or radiation plus esmeprazole groups. For the ensuing 2 weeks, tumor size was measured every day using standard caliper measurement, and the area of the tumor was calculated by multiplying the length and width of the tumor mass. Seven days after injection of the cancer cells, a second dose of radiation (10 Gray) was administered in the animals that received first dose of radiation. On day 28, all the animals were euthanized and the tumor mass in each animal was explanted for comparison, and for histopathological and immunohistochemical studies.

### Histology/immunohistochemistry

Tissue sections on charged glass slides were cut to 5 μm and deparaffinized in xylene and rehydrated via a stepped-gradient in ethanol. Peroxidase blocking, heat-induced antigen retrieval, and primary antibody incubation were performed per standard protocol under the following abbreviated conditions: anti-Ki67 rabbit monoclonal antibody (Cell Signaling; cat # 9129; 1:400), with citrate (pH 6.0) and SignalStain^®^ Boost IHC detection reagent. The primary antibody was incubated at room temperature for 1 hour followed by standard chromogenic staining with the Envision Polymer-HRP 3,3′diaminobenzidine (DAB; Dako) process. HRP-conjugated anti-rabbit secondary antibody (Cell Signaling; cat # 8114) was used to develop the signal. Qualitative immunohistochemistry scoring was performed by a pathologist who was unaware of the treatment that the animals received. All immunohistochemistry results were evaluated against positive and negative controls. Tissue sections from the same blocks were used for H&E staining using standard protocol.

### Statistics

The number of animals per study group was calculated using power and sample size calculation (PS; Vanderbilt University). One-way ANOVA (GraphPad prism; La Jolla, CA, USA) was used to analyze data and multiple groups were compared using ANOVA followed by Bonferroni posthoc test. Differences between two groups were compared using unpaired *t* test. All data are expressed as Mean ± SEM unless indicated otherwise. Differences are considered statistically significant at *p* value below 0.05 (*p* < 0.05).

### Study approval

The animal study was review and approved by Baylor College of Medicine’s Institutional Animal Care and Use Committee (IACUC # AN-7127). All other reagents used in this study are from commercial sources.

## SUPPLEMENTARY MATERIALS





## Abbreviations

FDA: Food and Drug Administration
HNSCC: head and neck squamous cell carcinoma
RPPA: reverse phase protein array
Cdks: cyclin-dependent kinases
