# Supplementary material for: Esomeprazole enhances the effect of ionizing radiation to improve tumor control
Source: Oncotarget. 2021 Jul 6;12(14):1339–53. doi: 10.18632/oncotarget.28008 (PMC8274720; doi:10.18632/oncotarget.28008)
Supplement: Supplementary file 1 [file oncotarget-12-1339-s001.pdf]

Esomeprazole enhances the effect of ionizing radiation to improve tumor control

SUPPLEMENTARY MATERIALS

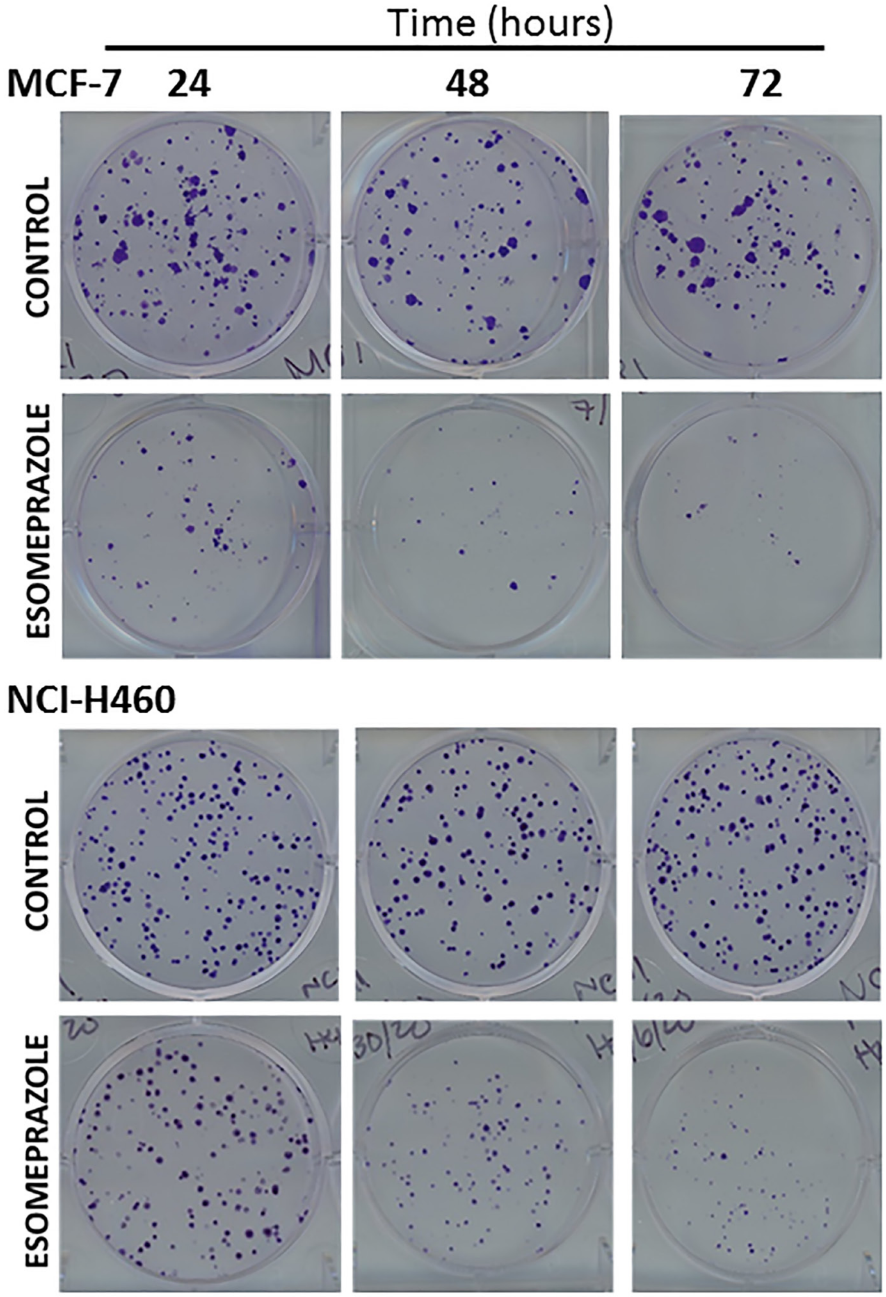

Supplementary Figure 1: Esomeprazole inhibits the growth of breast and lung cancer cells *in vitro*. Lung (NCI-H460) and breast (MCF-7) cancer cells were treated with control (water) or esomeprazole (200 µM) for up to 72 hours, and were allowed to form colonies for ensuing 7 and 14 days respectively. Crystal violet stained colonies are shown.

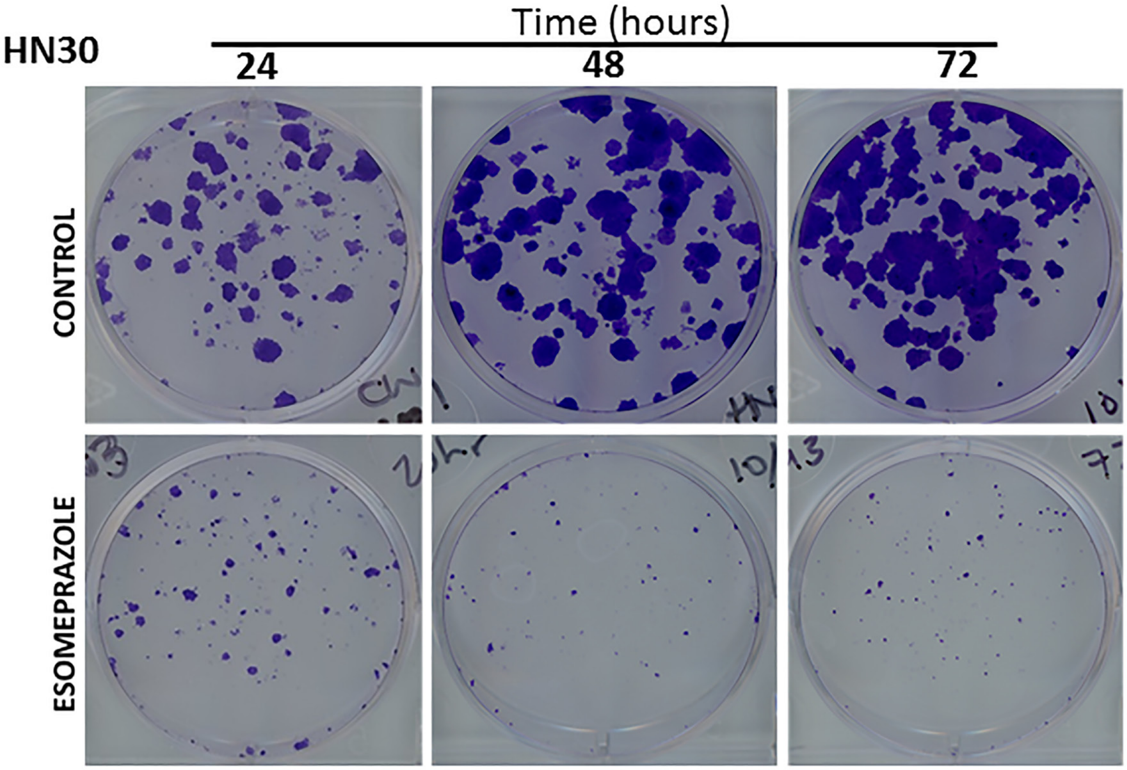

Supplementary Figure 2: Esomeprazole inhibits the growth of head and neck cancer cells in a time-dependent manner. Head and neck (HN30) cancer cells were treated with control (water) or esomeprazole (200 μM) for up to 72 hours, and were allowed to form colonies for 2 weeks. Crystal violet stained colonies demonstrate that the anticancer effect of esomeprazole increases with increased treatment time.

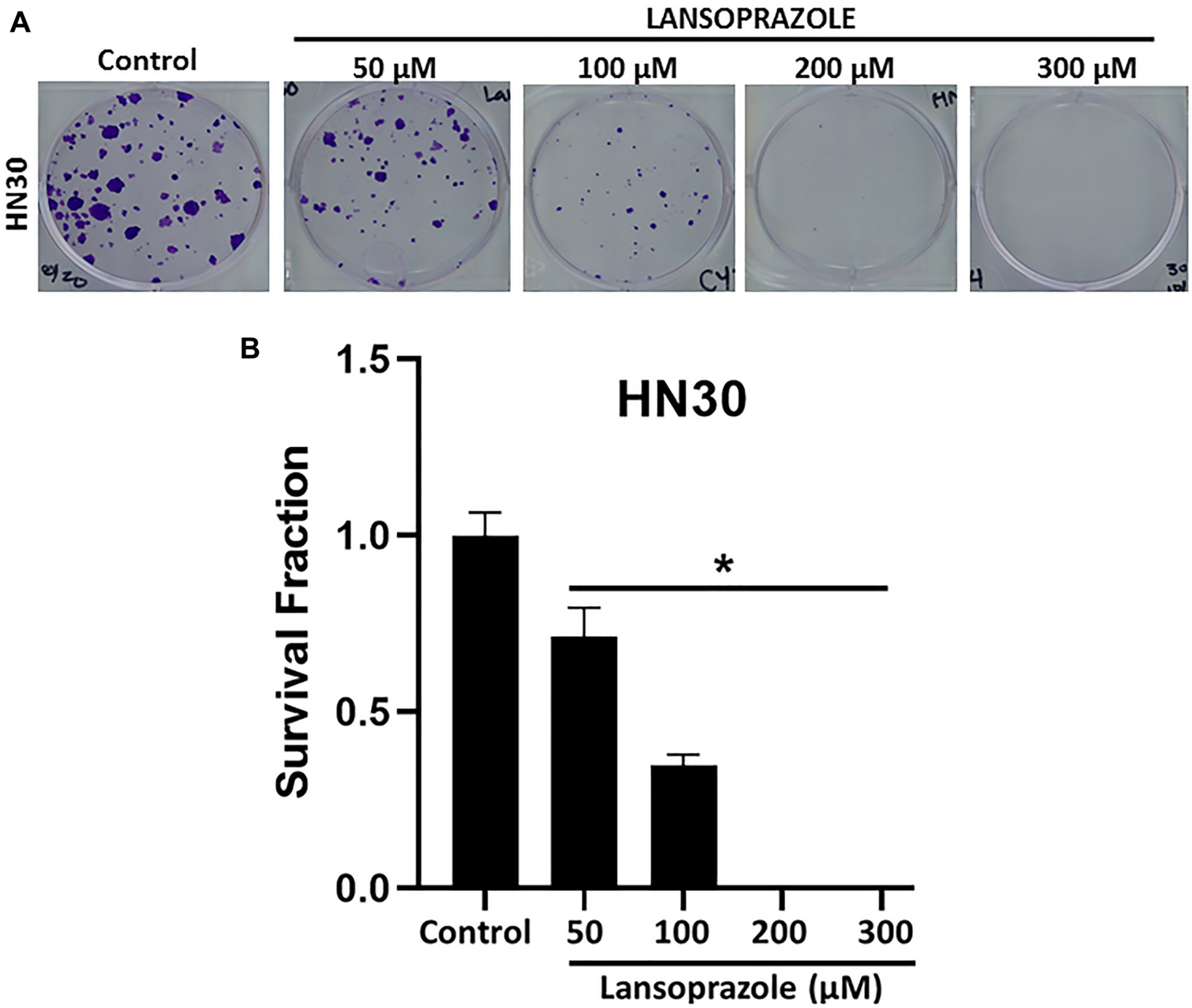

Supplementary Figure 3: Lansoprazole inhibits cancer cell growth. Head and neck (HN30) cancer cells were treated with control (water) or lansoprazole for 24 hours and were allowed to form colonies for 2 weeks. In (**A**) Crystal violet stained colonies show that the anticancer effect of lansoprazole increased with increased concentration of the drug. In (**B**) survival fraction of vehicle and lansoprazole treated HN30 colonies is shown. $^{*}p < 0.05$ vs control.

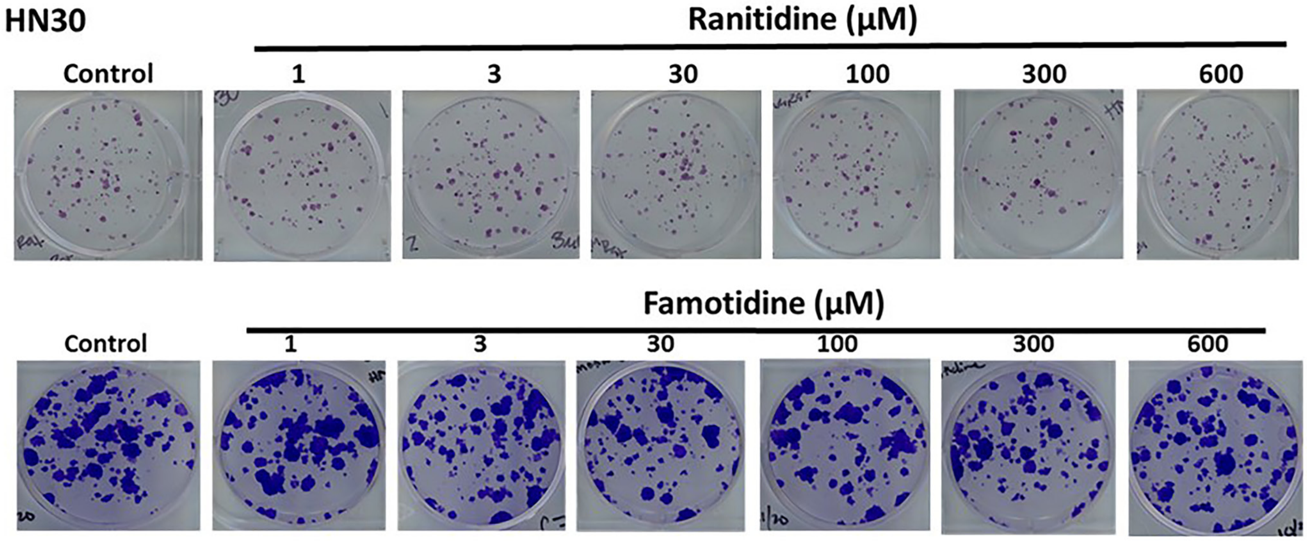

Supplementary Figure 4: Histamine 2 receptor antagonists (H2RAs) have no effect on cancer cell growth. Head and neck (HN30) cancer cells were treated with the H2RAs ranitidine or famotidine up to 600 µM for 24 hours and were allowed to form colonies for 2 weeks. Crystal violet stained colonies are shown in A (ranitidine) and B (famotidine).

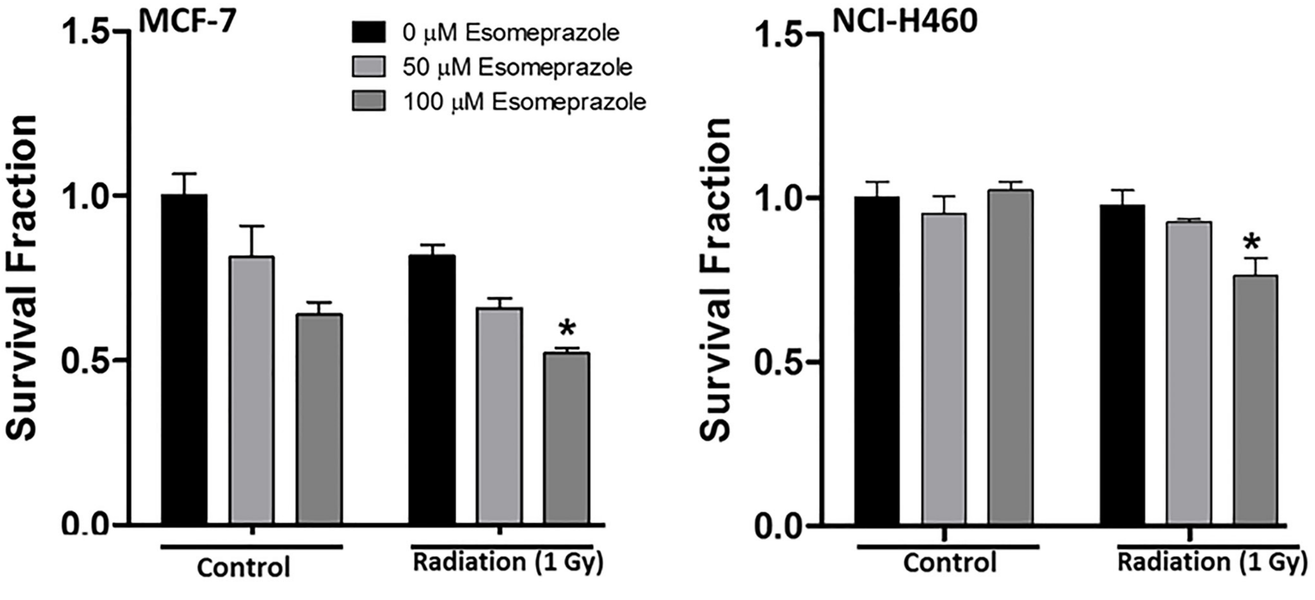

Supplementary Figure 5: Esomeprazole sensitizes breast (MCF-7) and lung (NCI-H460) cancer cells to radiation. MCF-7 and NCI-H460 cells were cultured in six-well plates. Some of the wells were subjected to ionizing radiation (X-rays; 1 Gray) in the absence or presence of esomeprazpole (50-100 µM for 24 hours). Cancer cell colonies were stained with crystal violet for comparison. The bar graphs show decreased survival fraction of MCF-7 and NCI-H460 cells following radiation and/or esomeprazole treatment in comparison to controls. Data is representative of four replicate experiments ($^{*}p < 0.05$ vs radiation only control).

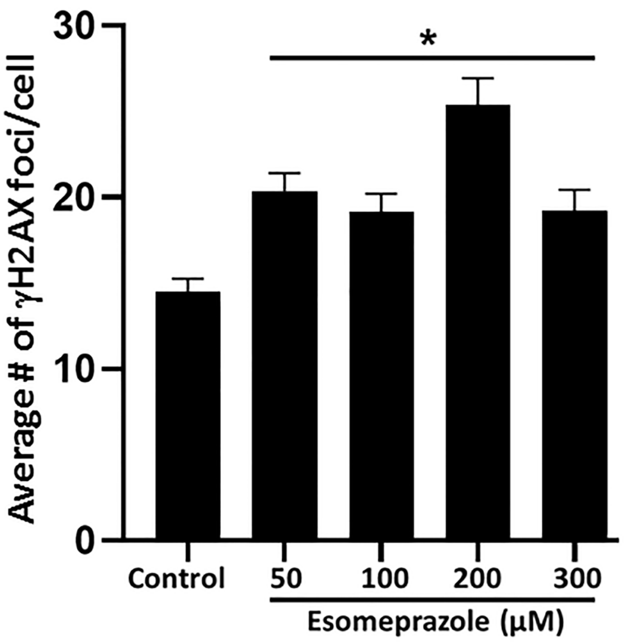

Supplementary Figure 6: Esomeprazole enhances radiation-induced DNA damage. HN30 cells were seeded in 384 well plates and exposed to radiation (X-rays; 1 Gy) in the absence or presence of esomeprazole. Cells were stained for the DNA damage marker γ-H2AX using phospho-histone H2AX (Ser139) rabbit monoclonal antibody and Alexa Fluor 488 conjugated goat anti-rabbit secondary antibody. Cells were imaged using the Optical Biosystems StellarVision (SV20) microscope and γ-H2AX foci were counted for comparison. Data is averaged from triplicate experiments. $^{*}p < 0.05$ compared to radiation only control.

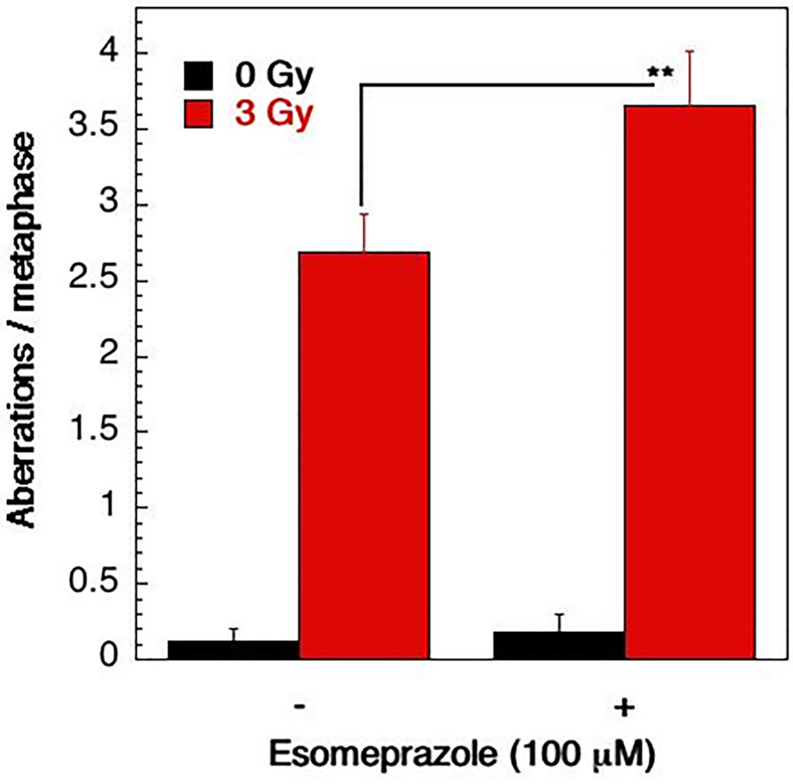

Supplementary Figure 7: Combination of esomeprazole with radiation enhances residual chromosome aberrations. Chromosomal abberations following exposure to ionizing radiation (X-rays; 3 Gy) in the absence or presence of esomeprazole was assessed in exponentially growing HN30 cells. G1-type of abrrations including dicentrics, fragements, breaks and gaps were quantified microscopically for comparison. $^{**}p < 0.05$ compared to radiation only control.

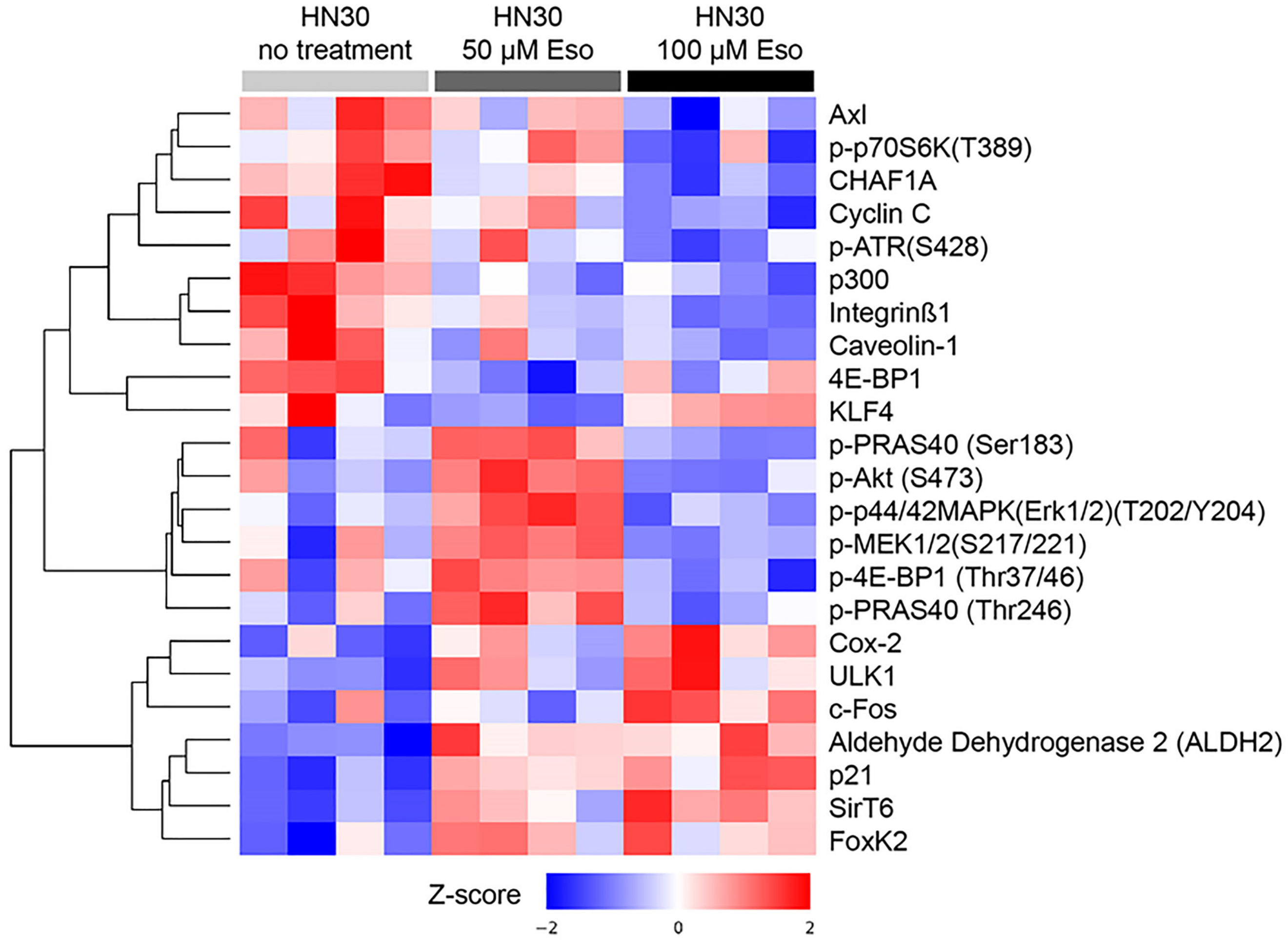

Supplementary Figure 8: Esomeprazole differentially regulates the expression of several cancer-related proteins. Heatmap generated from Reverse Phase Protein Assay (RPPA) assay showing HN30 proteins that are differentially regulated by esomeprazole. Each group was run in biological quadruplicates with technical triplicates. Data shown is for statistically different values at $p < 0.05$ and at least 1.5-fold difference.

A

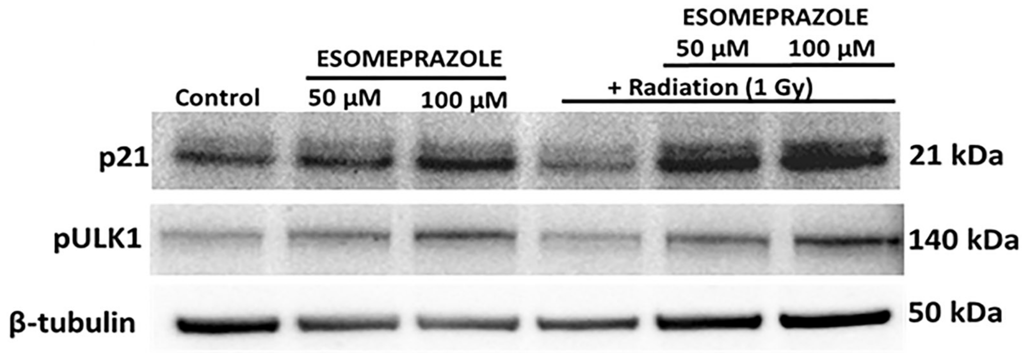

Rel Density						
p21	1.00	1.42	2.02*	1.00	1.33	1.30*
ULK1	1.00	1.65	2.60*	1.00	1.23	1.50*

B

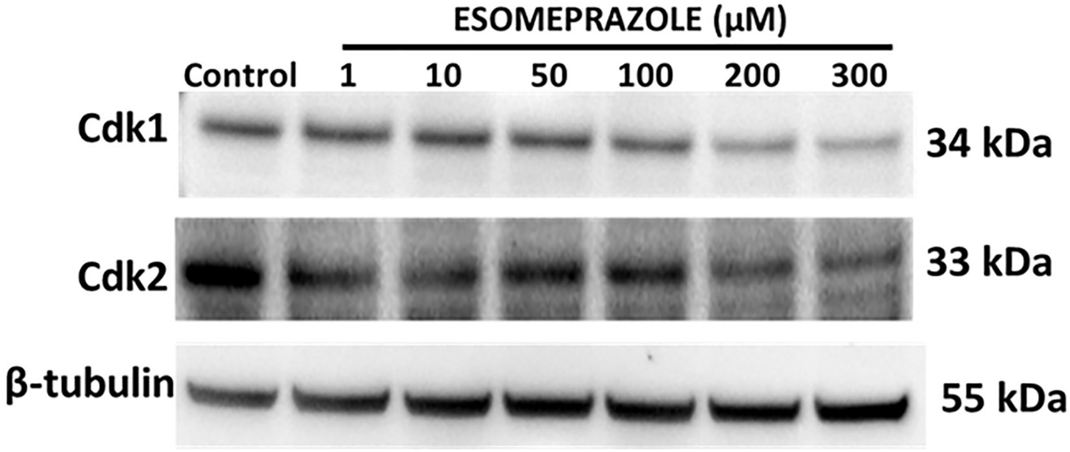

Rel Density							
Cdk1	1.00	0.88	0.68	0.62	0.58*	0.35*	0.25*
Cdk2	1.00	0.52*	0.29*	0.48*	0.48*	0.27*	0.21*

Supplementary Figure 9: Esomeprazole regulates several cell cycle-related proteins in HN30 cells. (**A**) upregulation of p21 and Unc-51 like autophagy activating kinase (ULK1); and (**B**) downregulation of Cdk1 and Cdk2. HN30 cells were treated with vehicle (water) or esomeprazole in the absence or presence of radiation (X-rays; 1 Gy) prior to harvesting cell lysate for Western analyses. Protein expression was probed using rabbit anti- p21,anti-ULK1, anti-Cdk1, and anti-Cdk2 antibody. Intensities of the protein bands relative to no treatment control are shown in the tables. $^{*}p < 0.05$ compared to vehicle control.

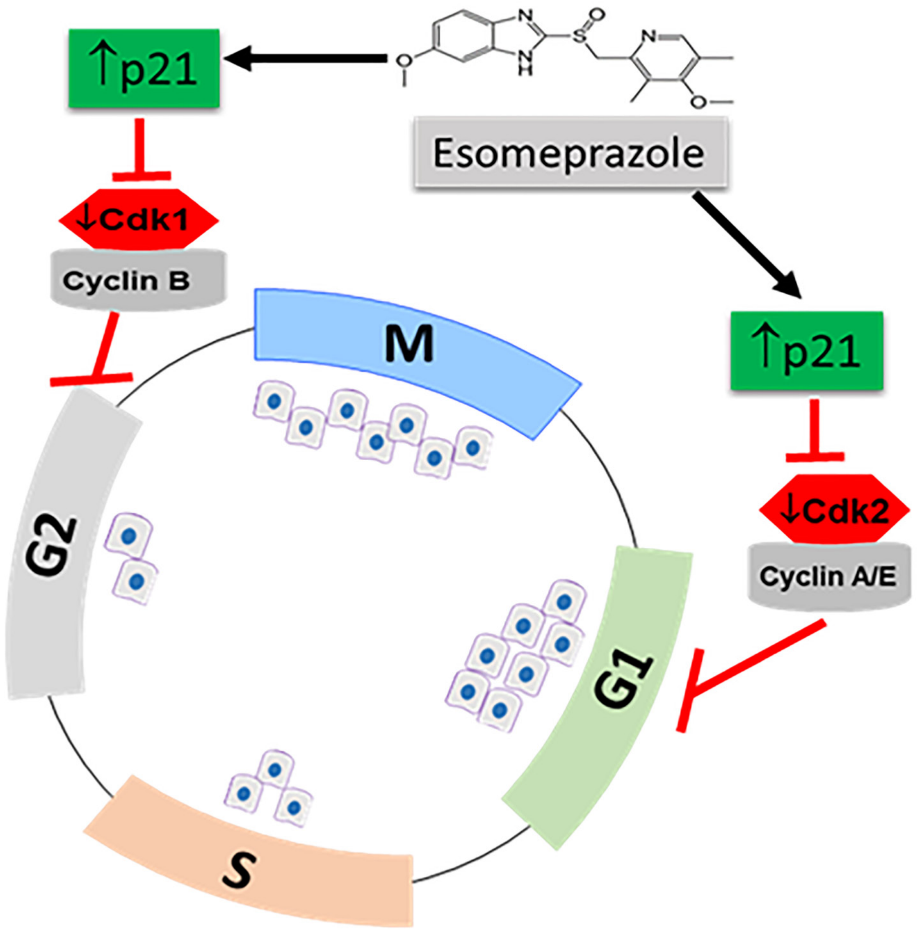

Supplementary Figure 10: Illustration of cell cycle regulation by esomeprazole. Upregulation of p21 protein by esomeprazole is expected to arrest cancer cells in the G1 phase of the cell cycle. Accordingly, fewer cells are expected to proceed to the DNA replication (S) phase of the cell cycle.

Supplementary Table 1: Esomeprazole differentially regulates several cancer-related proteins. See Supplementary Table 1
